# Hyperspectral hybrid method classification for detecting altered mucosa of the human larynx

**DOI:** 10.1186/1476-072X-11-21

**Published:** 2012-06-21

**Authors:** Ron Martin, Boris Thies, Andreas OH Gerstner

**Affiliations:** 1 Laboratory for Climatology and Remote Sensing, Faculty of Geography, University of Marburg, Marburg 35037, Germany; 2 Department of Otorhinolaryngology/Head and Neck Surgery, University of Bonn, Bonn, 53127, Germany

**Keywords:** Hyperspectral imaging, Signature extraction, Automatic target detection, Endoscopy, Tissue characterization, Mucosal surfaces, Laryngeal disorders

## Abstract

**Background:**

In the field of earth observation, hyperspectral detector systems allow precise target detections of surface components from remote sensing platforms. This enables specific land covers to be identified without the need to physically travel to the areas examined. In the medical field, efforts are underway to develop optical technologies that detect altering tissue surfaces without the necessity to perform an excisional biopsy. With the establishment of expedient classification procedures, hyperspectral imaging may provide a non-invasive diagnostic method that allows determination of pathological tissue with high reliability. In this study, we examined the performance of a hyperspectral hybrid method classification for the automatic detection of altered mucosa of the human larynx.

**Materials and methods:**

Hyperspectral Imaging was performed *in vivo* and 30 bands from 390 to 680 nm for 5 cases of laryngeal disorders (2x hemorrhagic polyp, 3x leukoplakia) were obtained. Image stacks were processed with unsupervised clustering (*linear spectral unmixing*), spectral signatures were extracted from unlabeled cluster maps and subsequently applied as end-members for supervised classification (*spectral angle mapper*) of further medical cases with identical diagnosis.

**Results:**

Linear spectral unmixing clearly highlighted altered mucosa as single spectral clusters in all cases. Matching classes were identified, and extracted spectral signatures could readily be applied for supervised classifications. Automatic target detection performed well, as the considered classes showed notable correspondence with pathological tissue locations.

**Conclusions:**

Using hyperspectral classification procedures derived from remote sensing applications for diagnostic purposes can create concrete benefits for the medical field. The approach shows that it would be rewarding to collect spectral signatures from histologically different lesions of laryngeal disorders in order to build up a spectral library and to prospectively allow non-invasive optical biopsies.

## Background

Spectral examinations using remote sensing data have proven to give insights on the distribution, structure and condition of different land covers [1]. Hyperspectral imaging improves accuracy and significance of such examinations [2] by providing the analyst with in-depth spectral data from the visible to the infrared bandwidth [3], thus overcoming the limitations of conventional imaging techniques. In practice, hyperspectral detector systems such as AVIRIS [4] and HYDICE [5] record radiation reflected from the ground in 200+ spectral channels from 0.4 to 2.5 μm simultaneously on each spot of a large array of spatial positions [6]. Advances made in recent years have also led to vast extensions of the possibilities for computational processing. Among the various methodologies for image analysis, such as anomaly detection [7] and material identification [8], the procedure of target detection reveals great advantage for pointed surface identifications [9]. Here the recorded scene is being scanned for a certain reflectance pattern (‘target signature’) that is known beforehand and that represents the spectral characteristics of a target surface in question. Thus, statements can be made on the appearance and distribution of certain vegetation [10-12], current levels of water stress [13] and pest infestation [14,15], morphology, soil topography [16] and other features.

In the medical field, much effort is spent on the development of reliable diagnostic techniques detecting pathological changes on tissue surfaces [17-19]. Thereby, diagnostic procedures are preferably as minimal invasive as possible to avoid interference with the patient’s tissue. Among the newer techniques, spectral optical imaging proves very promising for future applications [20-24]. Hyperspectral imaging in particular may provide a procedure that is less invasive than conventional diagnostic techniques and has potential for improved detection of abnormalities and pathological surface alterations [25,26]. Even though hyperspectral biomedical imaging has been well established on the technical side [27,28], corresponding applications often lack expedient classification procedures for adequate image analysis. The question, therefore, is whether additional benefits may be attained for the medical field when existing procedures taken from remote sensing applications are used to meet biomedical demands.

Both supervised and unsupervised classification procedures have been applied to hyperspectral surface detections of remotely sensed data [29,30]. In supervised learning, classification results highly depend on the number and quality of labeled samples [31]. Thus, errors can be minimized when the data is represented by a sufficient number of classes and class signatures are well separable in numerical feature space [32]. In unsupervised learning, spectral clusters and target areas are not necessarily coherent, but depend on the signature’s characteristics. Thus, final classes may not represent the actual regions of interest, even though unsupervised methods show insensitivity towards the dimensionality of spectral input [2]. An approach to meet both requirements - adequate end-member classes and potentially meaningful target assignments - is the so called *Hybrid Method*[31], which combines both supervised and unsupervised classification in a single procedure [32].

In this study, target detections using the hybrid method classification are performed on hyperspectral images of mucosal surface alterations of the human larynx. As no reference spectra for laryngeal disorders have yet been collected, the approach extracts spectral information from unsupervised clustering for subsequent supervised classification. Thus, there is no need to generate adequate training sites in every image scene, which would require the expertise of clinical experts with longstanding experience. The study is aimed at examining the principle applicability and performance of signature-based classifications to an automated detection of laryngeal disorders. In this way, the investigation is designed as a feasibility study that proposes a first attempt to assess the qualification of hyperspectral signatures for diagnostic detections. We hypothesize that pathological subregions of human vocal cords can be identified by means of unsupervised clustering, and that extracted hyperspectral signatures from unlabeled cluster maps can be applied to automatic target detections in second medical cases with identical diagnosis. Thus, analytical tools derived from remote sensing demands can be applied to demands of diagnostic recordings of suspicious tissue surfaces, providing an investigative attempt to treat hyperspectral data in medical studies and enabling further research on non-touch optical biopsies.

## Materials and methods

### Data acquisition

Five patients (cases #1-#5) with two histological findings (2x hemorrhagic polyp, 3x leukoplakia) were included in our study. Microlaryngoscopies were scheduled by arrangement and informed consent was obtained. Hyperspectral recording was performed *in vivo*. Endoscopies were realized using two different operation microscopes (OPMI Pentero and Vario, Carl Zeiss Meditec AG) under general anesthesia in jet-ventilation, as previously described in [33]. In order to generate monochromatic images at a consistent array of 10 nm wavelength steps, a Polychrome V (TILL Photonics) was synchronously triggered with a monochromatic high-resolution camera (AxioCam HRm, Zeiss MicroImaging). The camera and the Polychrome V light source were connected to both microscope types using a C-mount thread module and integrated optical fibres. Other than that, no additional changes to the standard operation setup were made, *i.e.* no further sampling or excisional biopsy was conducted. Subsequently, the resulting image stacks were cropped by narrowing down the field of view to the relevant parts of the vocal cords. Here the aim was to generate images of homogeneous illumination that show pathological tissue changes on the vocal cords in a purely biological environment. This is due to the fact that illumination was not homogeneous in all recorded stacks and that technical parts of the endoscope often protruded into marginal areas of the images.

### Data processing

The general approach of hybrid method classification proceeds by segmenting the image stack into *n* clusters, creating a map of unlabeled cluster segments and subsequently using hyperspectral signatures from unsupervised learning for final supervised classification [32]. In our study, the unsupervised clustering used a combined process of signature development and spectral unmixing based on the *Linear Mixture Model* (LMM). A wealth of hyperspectral examinations previously applied LMMs and demonstrated good results for improved determinations of surface components [34-38]. Since the basic assumption of the LMM is that the reflection value of a pixel is a combination of the signature means of all components present in the pixel, the expected reflection value *R* of that pixel covered by various ground categories can be expressed by the formula

(1) $$R=f_{1}\mu_{1}+f_{2}\mu_{2}+\ldots +f_{N}\mu_{N}$$

where *f *= proportion of the *n*th surface component (*n* = 1,…, *N*), *μ *= expected spectral signal from the *n*th surface component*,* and *N* = total number of components [39]. The applied clustering approach aimed to provide a hierarchical order of the ‘strongest’ spectral signatures present in the image scene. Therefore, a first pixel was identified whose reflection value was the highest among all pixels present. Following equation (1), this is the pixel whose sum of squares of all reflection values equaled the maximum numeric value of all pixels. The reflection values of that pixel in all channels were extracted and subsequently applied as an end-member signature^a^ for cluster 1. As it is an *internal criterion* of spectral clustering [40], the unsupervised clustering approach further aimed at providing this hierarchical order of spectral signatures with the highest possible separability in numerical feature space^b^, thus preparing signatures for end-member subsets with high intra-cluster similarity and low inter-cluster similarity [41]. The second signature was therefore chosen by finding the pixel with reflection values in the respective channels that were most different from those of signature 1 in numerical terms. To this end, the reflection values in the respective channels were subtracted from those of the corresponding channels of signature 1. The resulting differences were squared and summed in order to identify the highest calculated value. The set of reflection values corresponding to the highest value described highest possible separability from signature 1 and thus was accepted as the end-member signature for cluster 2.

In our study, this procedure continued until a predefined number of 20 signatures was generated for all five image stacks. This relatively high number was chosen in light of the subsequent classification processes whose quality of data representation depends significantly on a sufficient number of spectral classes. Furthermore, the number resulted from our empirical experience. While clusters from 1 to 20 revealed at least one class with notable correlation to altering tissue locations, a cluster number of over 20 revealed clusters of increasingly useless information. In order to develop unlabeled cluster maps, *posterior probabilities* were calculated for each pixel to belong to one of the 20 classes. A comparison of probabilities yielded the maximum likelihood for each pixel’s affinity and classes were assigned accordingly. As the approach of the hybrid method classification was aimed at automatic target detections of pathological tissue expansions on human vocal cords, resulting clusters from unlabeled cluster maps were compared to abnormal surface expansions on the corresponding white light images with the support of clinical experts and operation protocols. This means that a visual interpretation by ENT (Ear-Nose-Throat) specialists and an inclusion of medical records was prompted to undertake a first assessment of the cluster locations. By highlighting the suspicious subregions of vocal cords as single spectral clusters, these clusters already gave an indication of the applicability of hyperspectral classifications to the applied mucosal surface recordings, even though the general qualification of the procedure has already been proven for previous recordings of that same area [33].

The final supervised classification aimed at automatic target detection was performed exemplarily for one diagnostic pair in each of the 2 histological findings. Therefore, unlabeled cluster maps of case #1 and #3 served as training sites to extract spectral signatures for all 20 classes from the respective 30 channels. The acquired end-members of case #1 were then applied to the final supervised classification of case #2 among the hemorrhagic polyps, and those of case #3 were applied to the final supervised classification of case #4 among the cases of leukoplakia. The *spectral angle mapper* algorithm was chosen for mapping spectral similarity between image spectra and cluster spectra in order to perform supervised classification. The algorithm is a well established similarity measure in hyperspectral classifications [42-44]; it treats reference and field spectra as vectors in *n*-dimensional feature space, with *n* corresponding to the number of bands and different vector positions representing differences in illumination for a target material. Similarity between spectra is then determined by calculating the resulting angle between regarded vectors. As the angle between both spectra remains constant for all examined positions [45], the approach offers the advantage of neglecting disturbances caused by light/shadow contrasts.

As the results of the automatic target detections in case #2 and #4 corresponded to a common two-group outcome (pathological/non-pathological), a reclassification of the 20 clusters to a binary decision map delivered the diagnostic expression for both detections. The value 1 (positive result) corresponded to the ‘pathological’ condition, while the value 0 (negative result) corresponded to the ‘normal’ condition. To perform a statistical validation, confusion matrices were then calculated as described in [46]. In our case, designated pixels from detection outcomes were compared to reference masks that illustrated the known diagnosis by defining the exact location of the altered tissue in cases #2 and #4 (corresponding masks were created with help of ENT expert knowledge). For assessment of the diagnostic accuracy, the traditional measures of *sensitivity* and *specificity* were calculated. As the sensitivity of a test refers to its capability to identify positive results [47], it calculated our model's ability to correctly detect pixels of pathological tissue. Specificity, on the other hand, refers to a test’s capability to identify negative results and calculated our model's ability to correctly diagnose pixels of healthy tissue. In addition, the *bias* was calculated to quantify possible over- or underestimations in our detections. Since the bias is the total number of positively detected pixels divided by the total number of truly positive pixels according to the reference mask, an ideal bias has the value of 1. A bias > 1 indicates an overestimation, whereas a bias < 1 indicates an underestimation [48].

## Results

### Imaging procedure

The hyperspectral imaging workstation was set up without technical problems. Both microscope types could readily be connected to the Polychrome V light source and the high resolution camera by applying the C-mount connector. The respiration stop during jet ventilation for movement reduction of the larynx (30 sec) posed no anesthesiological problem for any of the patients. The hyperspectral imaging procedure provided hyperspectral image stacks containing 30 spectral bands from 390 to 680 nm for all cases. Each image stack could be treated as a series of channels that were associated with identical pixel locations. This enabled further performance of hyperspectral routines, whereby the image stacks represented collections of various, evenly spaced wavelength bands recorded for one region of interest.

### Unsupervised clustering

**Figure 1:**
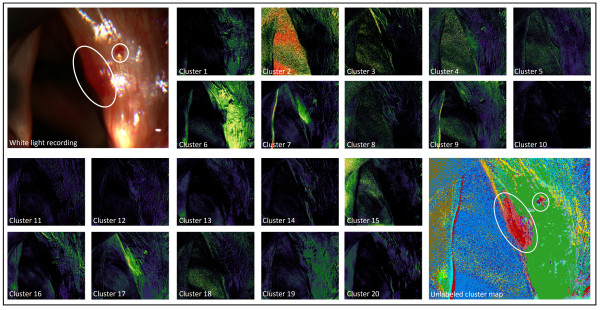
**Unsupervised clustering of case #1.** Top Left: White light image with marked pathological tissue locations (in accordance with expert knowledge). Middle: 20 class images from spectral unmixing. Bottom Right: Unlabeled cluster map with red colored classes (7 and 17) showing notable accordance with the expansion of the hemorrhagic vocal cord polyp.

**Figure 2:**
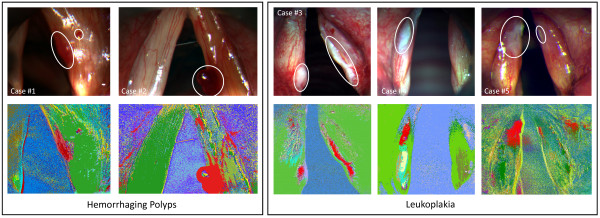
**Unsupervised clustering of all cases examined.** White light images with marked pathological tissue locations (in accordance with expert knowledge) (top) and unlabeled cluster maps (bottom), sorted by diagnosis. Red classes show notable correspondence with the expansions of altered tissue.

**Figure 3:**
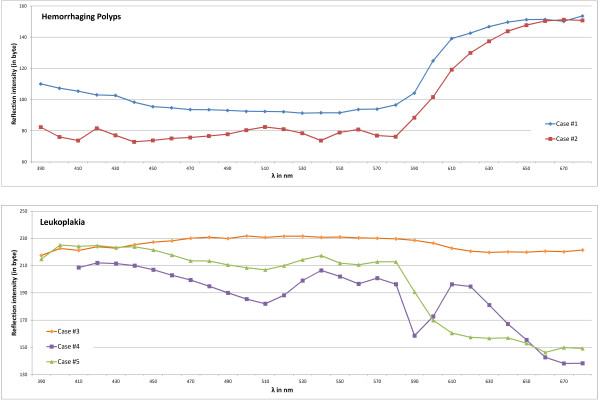
**Spectral profiles of pathological cluster locations for all cases examined.** Mean reflection values were calculated and visualized for all spectral bands of the pixels present within the cluster locations.

Unsupervised clustering by means of linear spectral unmixing delivered 20 class images, each presenting the degrees of affinity for the pixels to belong to their class image’s superordinated signature. Due to the mathematical principle of a maximum difference between the classes, the images demonstrated strong heterogeneity in their classes’ composition, thus uncovering clusters with different peculiarities. Unlabeled cluster maps subsequently visualized cluster expansions in one image, which enabled medical comparison with the white light images. Figure 1 exemplarily shows the white light image, unmixed class images, and unlabeled cluster map for case #1 (recorded from 390 to 680 nm with an image size of 368x322 pixels each). For better recognition, those classes identified as having the greatest correspondence with altered mucosa locations were colored red within unlabeled cluster maps. In all of the resulting cluster maps, either one or two spectral clusters highlighted areas with notable correlation with locations of pathological surface tissue (see Figure 2). Spectral profiles of these matching clusters were attained by calculating mean reflection values for all bands, thus visualizing the averaged spectral characteristics for all leukoplakia and hemorrhagic polyp incidences examined. In cases #1 and #2, profiles showed similarity in their general course, with almost constant reflections from 390 to 580 nm and a flattening increase from 580 to 680 nm (see Figure 3.). Except for reflection at 670 nm, altered tissue of case #1 demonstrated higher reflection values in all bands. In cases #3, #4 and #5, similarities could be found in reflection trends from 390 to 430 nm. Cases #4 and #5 also show a parallel reflection course from 390 to 590 nm. Other than that, profiles differed both in shape and numerical terms.

### Supervised classification

**Figure 4:**
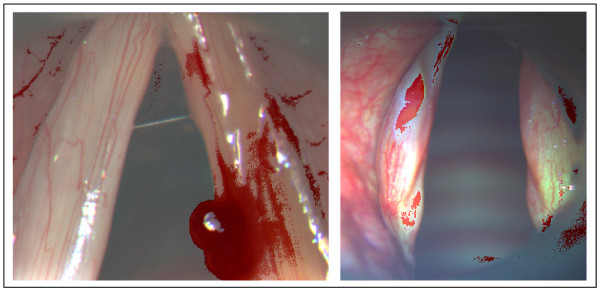
**Hybrid method classification applied to automatic detection of the hemorrhagic polyp in case #2 (left) and the leukoplakia in case #4 (right).** A discoloration of negligible classes highlights the clusters (red) that represent the signatures matching the pathological surface tissue. White light images were placed beneath the target detections to aid manual recognition.

Extracted signatures from unlabeled cluster maps were successfully applied as end-member spectra for final supervised classification of second medical cases with identical diagnoses. The results delivered classification maps showing the pixels of cases #2 and #4 assigned to spectral classes from the external signatures of case #1 and #3. Corresponding classes were colored red and negligible classes were erased in order to more easily recognize the spectral clusters that previously highlighted altered tissue surfaces in the initial cases. In addition, white light images were placed beneath the classification results by means of a transparency effect. Target detections showed strong correlation of considered class locations with pathological tissue expansions in second medical cases with identical diagnoses (see Figure 4).

Confusion matrices delivered different results for the detections in the two diagnoses. The outcomes for both categories (pathological, normal) are shown in Table 1. The statistical validation unveiled a sensitivity of 87.41% and a specificity of 97.91% for the detection of the vocal cord polyp. For the detection of the leukoplakia a sensitivity of 53.61% and specificity of 99.21% was derived. Thus, in both cases the hybrid method satisfied with a probability of over 97% of correctly classifying normal tissue. On the other hand, the approach shows a markedly higher probability to correctly identify the altered tissue of a vocal cord polyp than to correctly identify the leukoplakia tissues. In case #2, the detection tended toward a slight overestimation of the pathological expansion by delivering a bias of 1.1796, whereas in case #4 the bias of 0.7565 implied an underestimation of the present leukoplakia.

**Table 1:** Confusion matrices with sensitivity/specificity analysis and calculated bias for the automatic target detections in case #2 and #4

**Automatic target detection in case #2**				**Automatic target detection in case #4**			
		**Pathological**	**Normal**			**Pathological**	**Normal**
**Reference Mask**	**p**	5627	811	**Reference mask**	**p**	1680	1454
	**n**	1967	92320		**n**	691	86808
sensitivity/specificity: 87.41%/97.91%				sensitivity/specificity: 53.61%/99.21%			
bias: 1.1796				bias: 0.7565			

## Discussion

Crucial differences exist between given circumstances in remote sensing applications of hyperspectral imaging and those of biomedical uses. Hyperspectral imaging in satellite observation utilizes the sun as light source, providing the target area with the entire spectrum of electromagnetic radiation. Spectral separation of the reflected energy for analysis is then performed during the recording procedure. In biomedical applications, crucial changes in the testing setup have to be made in order to facilitate adequate illumination of a biological region of interest. In the context of this study, the larynx is a straitened area of the human body with no natural sunlight interfering. These circumstances necessitate the need to implement refractors, monochromators or tunable filters to separate the light into single bands. Thus, illumination was performed exposing the larynx by means of a monochromatic light source. Also, no additional filter was desired between the mucosa and the camera, since illumination was limited to a confined number of reflecting photons in our test setup. Eventually, adequate conditions could be established by means of monochromatic illumination and synchronously triggered high-resolution imaging, allowing modern medicine to perform hyperspectral imaging and opening doors to potentially benefit from existing data processing techniques derived from the field of earth remote sensing.

In this study on the application of hyperspectral hybrid method classification to automatically detect laryngeal disorders, good results could be achieved throughout all utilized procedures. The results indicate that areas of hemorrhagic vocal cord polyps and present leukoplakia can be recognized as independent elements of hyperspectral image compositions using unsupervised image clustering. We can thus infer a considerable separability of pathological mucosa from environmental tissue with an unexpectedly high degree of accuracy. Because no additional input of reference spectra was applied, class matches were attained *automatically*. This also speaks for the clustering processes’ capability to diagnose abnormal tissue changes as being *divergent* from surrounding mucosa. No considerable difference could be perceived in the quality of recognition between the different histopathological groups. However, spectral profiles from clusters covering pathological tissue locations delivered notable differences. While in cases #1 and #2, profiles were still alike in their general course throughout all channels, similarities in shape among the cases #3, #4 and #5 could only be found for certain segments of the entire spectrum. In numerical terms, cases clearly differed in both histopathological groups. This particularly applied to the three cases of a present leukoplakia between 590 and 680 nm. In view of the region examined, these variabilities in spectral response may be ascribed to the specific manifestations of a disease that vary from patient to patient. Even though significant spectral similarities should be assumed for altered surface tissue with identical diagnoses, spectral responses will accordingly vary due to natural pathogenetic peculiarities. Further, differences in the anatomical environment of the larynx caused differences in illumination during hyperspectral recording in all cases.

Final supervised classifications unambiguously assigned pixels of second medical cases #2 and #4 to the signature classes from unlabeled cluster maps of initial cases #1 and #3. With special regard to the classes matching with local alterations in the initial stacks, results surprised by showing primarily those pixels being ascribed, which represented the core area of the second case’s *own* pathological expansion. Even though according classes also spread into further areas of the view frame, mistakenly assigning few locations of healthy tissue to the ‘pathological’ signatures, the centers of pathological expansions were identified precisely. Thus, only minor failures distort the result of an accurate and locally correct diagnosis in both cases as confirmed by statistical validation. Even though a notable difference could be assessed in the sensitivity of both detections, the training set of 2 patients is too small to draw conclusions on the methods’ qualification on different diseases yet. However, the results are particularly meaningful in view of the above mentioned spectral variability of the examined abnormality appearances. At that point, measuring spectral similarity between the target and the pixel spectra by means of the spectral angle mapper appeared to be a useful choice. It improved classification results by disregarding specific surface irregularities of the vocal cords that naturally led to inhomogeneous reflections in every patient. Consequently, it ascribed identical mucosa alterations in both cases to the same spectral cluster and thus, coped the problem of spectral variability of equal surface components notably well, which remains a major challenge for classifications and target detections in hyperspectral imagery.

These results show that the initially postulated hypotheses that pathologic subregions of human vocal cords can be identified by means of unsupervised clustering and that extracted hyperspectral signatures from unlabeled cluster maps can be applied in a practical context to automatic target detections in second medical cases of identical diagnosis can be confirmed. Furthermore, the overarching question of this study whether the medical field can profit from taking existing procedures from the field of remote sensing and applying them to biomedical demands, where imaging modalities are already able to deliver corresponding image stacks, can be answered *affirmatively*.

## Conclusions

The insights attained on the performance and potential applicability of hyperspectral classifications for the automatic detection of laryngeal disorders may give an impetus for further research on hyperspectral classifications in medical usage. The underlying reason is that laryngeal disorders of the vocal cords cause specific changes in the surface structure of the covering epithelium. These alterations, often expressed by atypical modifications of color and texture, pave the way for spectral determinations using automatic target detections. This study was performed exemplarily for five image stacks featuring two different histopathological findings of benign alterations. As it is known that carcinoma of the vocal cords significantly contrast with their non-pathological environment likewise (albeit with a higher variability of spectral characteristics), similar results may be expected for malignant proliferations. More specifically, carcinoma of the human larynx should be equally identifiable by means of hyperspectral clustering procedures, while an establishment of automatic target detections may require a larger set of reference spectra, as well as proper calibrations of statistically suitable similarity measures, to compensate for spectral variability. However, this potential is particularly relevant for the progress of cancer diagnostics and shall be the subject of upcoming investigations.

Given a sufficient number of clinical cases, spectral signatures can be collected for histologically different lesions of laryngeal disorders in order to build up spectral libraries covering a wide variety of clinical findings. More complex classification procedures may then be applied that are specifically designed for use with library spectra and will improve detection accuracy by taking into account the severity and stage of a disease. Consequently, first attempts of clinical trials would be allowed, making an essential step towards the establishment of non-touch optical biopsy.

## Endnotes

^a ^The term end-member signature denotes the spectral response pattern of the pixel that defined the class metrics of a certain cluster for spectral unmixing.

^b ^In this case, numerical feature space is an abstract space with a feature number equal to the number of spectral channels. Each signature is a defined position in 30-dimensional space, which enables numerical quantification of their separability.

## Competing interests

The authors declare that they have no competing interests.

## Authors’ contributions

RM edited the data sets, selected the classification approach and performed subsequent image processing. RM also conducted the core literature review, and conceived and drafted the manuscript with contributions from BT and AOHG. BT supervised the preparation of the data sets, the image processing, and the interpretation of classification results. AOHG performed microlaryngoscopies for hyperspectral imaging, provided expert knowledge on histopathological backgrounds and gave hints on the medical side for data interpretation. RM, BT and AOHG jointly conceived the design of this study. All authors read and approved the final manuscript.
